# In Memory of David Charles

**DOI:** 10.1155/S1064744994000396

**Published:** 1994

**Authors:** Sebastian Faro


Infectious Diseases in Obstetrics and Gynecology 2:49–55 (1994)
© 1994 Wiley-Liss, Inc.

### In Memory of David Charles

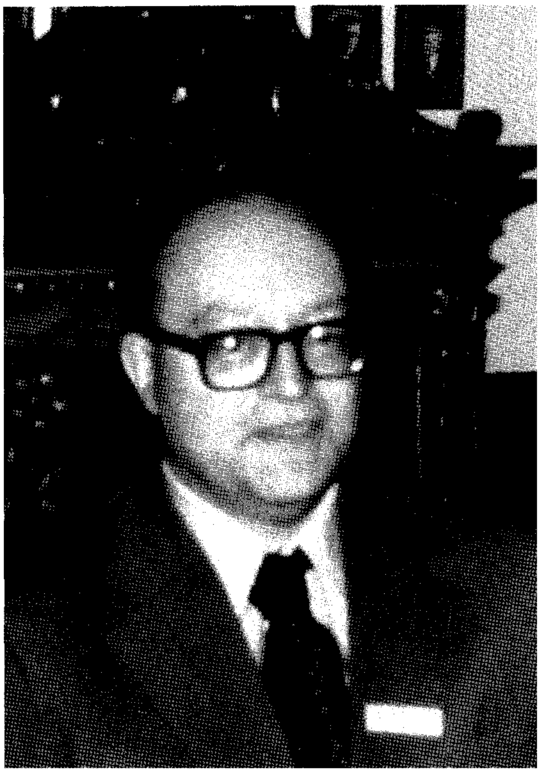

David Charles

This issue is dedicated to the memory of David Charles, who died on August 2, 1993, leaving a void affecting many academicians and clinicians in obstetrics and gynecology throughout the world.

For those of you who may not have known him, David was Welsh by birth and educated in London. He held certifications in obstetrics and gynecology from the Royal College of Obstetricians and Gynaecologists, the Canadian College of Obstetricians and Gynecologists, and the American Board of Obstetricians and Gynecologists. Aside from some time spent in Canada, he resided in the United States for many years. He chaired the Departments of Obstetrics and Gynecology at Boston University School of Medicine, Memorial University of Newfoundland, and Marshall University School of Medicine. His accomplishments include, most notably, his active role as a founding member of the Infectious Disease Society for Obstetrics and Gynecology. Among the honors bestowed upon him were membership on the Canadian Council of the Royal College of Obstetricians and Gynaecologists, presidency of the Infectious Disease Society for Obstetrics and Gynecology, and membership on the American Representative Committee of the Royal College of Obstetricians and Gynaecologists. His work and publications, particularly related to infectious diseases of pregnancy, have made an enormous contribution to our knowledge and advancement in this area.

I would like to thank each of the contributors as well as Nancy McComas of Marshall University School of Medicine for making this memorial issue possible. Each contributor has prepared an article for this issue as a tribute to the work and accomplishments of David Charles. Here are their expressions of appreciation and memories of David Charles, the physician, the academician, and the man.

*Sebastian Faro, M.D., Ph.D.*
*Editor-in-Chief*

I first learned about David from his writing and editing. For me, those articles established his credibility as a scientist and clinician, so, at our first meeting, I knew who he was. In 1973, David and Gilles Monif invited me to breakfast in an attempt to convince me to become a member of a new formal scientific society within obstetrics and gynecology devoted to infectious diseases. Although I was a bit skeptical, thinking that it would be just another "dog-and-pony" show or a vehicle for pharmaceutical promotion, David's stature and enthusiasm convinced me otherwise. His vision and that of the other founding members of the Infectious Disease Society for Obstetrics and Gynecology have been fully realized.

In the succeeding 15 years, through the Society and correspondence, my wife, Freddie, and I became very friendly with David and his wife, Jean, because of similar cultural and nonscientific likes and dislikes. We tried frequently to coordinate travel and leisure time together, but the exigencies of our lives and careers never allowed that—something for which we are truly sorry.

In 1984, David became my sponsor for membership in the Infectious Disease Society of America (without my knowledge). In 1985, he became my sponsor for membership in the American Gynecological and Obstetrical Society. His encouragement of me to achieve these goals is only a small example of David's behavior. He, as a matter of course, took it upon himself to promote and encourage people within our specialty in the true sense of the word "mentor." Unfortunately, there are too few like him who look to the future of people and of our specialty.

David would have responded to my article with a proper British accent, "Indeed!" In "Americanese," that would be "Right on!" David was as much a scientist as he was a clinician. His approach to an infectious disease that is a threat to the public health would be a scientific one: Who is infected? How did they get infected? How can we prevent this? Taking a political course against something that is so obviously in the public-health domain is not medically correct—even if it is politically correct. The abortion fiasco in Boston in the early 1970s is an example of David's lack of tolerance for politics replacing medical judgment. David, fussing and fuming at a state health commissioner, would declare that HIV infection is a sexually transmitted disease!

*Marvin S. Amstey, M.D.,*
*Professor*
*Department of Obstetrics-Gynecology*
*University of Rochester Medical Center*
*Rochester, New York*

I met David Charles at the first meeting of the Infectious Disease Society for Obstetrics and Gynecology in Gainesville, Florida, in 1974. We quickly recognized that we both enjoyed taking long walks and together set off along the roads through the swampland surrounding Gainesville in search of alligators. Our desire was to see the alligators before they saw us. On that walk and on many subsequent jaunts with David, I developed an intense affection and admiration for him. I loved his wit, breadth of knowledge, warmth, enthusiasm, and provocativeness. I was enchanted by his accent and command of language. David was entirely open and honest. I recall him writing me some of the most cherished and complimentary letters I have received, but I also recall him giving me hell for saying things about which he felt less kindly. In my office, I have a photograph of David (taken at the Tubingen meeting in 1979) next to the photograph of the other Society presidents taken at the Society meeting in August 1993. (It is poignant that David died just a few days before the 1993 meeting.) David is smiling and seeming to say to me, "Pursue excellence in all you do."

In addition to all of David's other attributes, I also found him to be totally unpredictable. My hope is that he would have considered our article to be a thoughtful and timely review of a pertinent subject and that the new information in the article provided insight into the human problem of preterm birth. On the other hand, David might just say that the article needed work on its prose and that any work done in animals required a huge leap of faith before application to humans.

*Ronald S. Gibbs, M.D.*
*Professor and Chairman*
*Department of Obstetrics and Gynecology*
*University of Colorado*
*Health Sciences Center*
*Denver, Colorado*

Those who were well acquainted with David Charles and counted him as a friend were saddened at his death in August of last year and miss him greatly. I first met David at an infectious disease meeting and was impressed that, in addition to his clinical acumen, he always valued the role of basic scientific inquiry and its application to disease processes. When he became the founding chairman of the Department of Obstetrics and Gynecology at Marshall University School of Medicine, he showed in a very tangible way his dedication to basic research by hiring a Ph.D. as the third faculty member of his department. In these early days, he was an obstetrician in 2 senses: he delivered infants day and night at 2 different hospitals, but spent much of his remaining time overseeing the delivery of an "infant" department. The latter was a high-risk pregnancy and, more often than not, seemed to be a breech presentation. During this time, he worked tirelessly with medical students (one-third of the first graduating class applied for residencies in obstetrics and gynecology), wrote an extensive syllabus, lectured, conducted hospital teaching rounds, developed an active private practice, maintained a grueling surgical schedule, assumed night-call responsibility with deliveries at all hours, and doused the political brush fires that abounded in the nascent medical school. But all of this was not enough. He was determined to fulfill the mission of rural health care by establishing several outreach clinics in remote areas. He accomplished this mission by personally driving winding back roads, with 1 or 2 medical students in tow, to clinics where frequently 60 patients awaited care in such unappealing surroundings as a county-courthouse basement. But this was still not enough. He continued a busy schedule of scholarly writing, traveling, lecturing, and, as he often called it, "Preaching the gospel of Marshall University." As a young member of his faculty, I was amazed at his stamina, watching him do twice the work of a person half as old.

On a personal level, he was an individual who filled the room with his presence and could not be ignored. Those who encountered him only casually felt overwhelmed by him. He was not amused by indolence or incompetence and was never one to "suffer fools gladly." But his true character was one marked by generosity and eagerness for the success of his faculty and staff associates. Interacting with him daily, I could not help but be impressed with his sense of humor, which often could only be described as insouciant playfulness. His very active professional life was balanced by his exuberant love of the arts and all of the wonders of life on earth that he shared in full measure with his devoted wife, Jean. I count it a genuine privilege to have had the pleasure of the company and collegiality of David Charles and will always treasure my memories of him.

*Bryan Larsen, Ph.D.*
*Professor*
*Departments of Microbiology and Obstetrics and Gynecology*
*Marshall University School of Medicine*
*Huntington, West Virginia*

David Charles was a friend bigger than life. I will give you a few vignettes to show you what I mean. My first recollection of speaking with David on a one-to-one basis came at a British obstetrics-gynecology congress in the late 1960s. It was the first trip to Europe for an insecure assistant professor from Michigan with no academic reputation. At the time, I was neither a member nor had I been invited to an SGI meeting. My talk on cervical dilatation graphs of women in labor was an innovative clinical study, but not earth shattering scientifically. David, a professor and chairman, befriended me. I was immediately aware of his size. He did not tower over me physically, for I am a big man, but, in fact, I felt he did. It was not a physical dominance but more as a protective older brother. I was struck by the fact that he had time to talk with me and I very much appreciated his words of encouragement. I next focused on David with his editorship of a textbook published by Lea & Febiger, *Obstetric and Perinatal Infections*. This text was published before its time, as it sold fewer copies than it should have because American obstetrician-gynecologists at the time of the book's release had not yet comprehended that infection was both important and relevant. In the early 1970s, David and I became close friends after he invited me to Boston to speak at an infectious-disease course. What a thrill! I met Maxwell Findland, a long-time infectious-disease idol of mine, and David and his wife, Jean, were a perfect host and hostess. After that, David and I became thick as thieves as we worked together with the other contributors of this memorial issue to form the Infectious Disease Society for Obstetrics and Gynecology in the United States. David was unique and refreshing. He was a searingly honest person who would not hesitate to tell anyone if he were disappointed in that person's study methods. His critiques were never voiced in private as gossip, but expressed openly as from one friend to another. Even when you were the subject of his critique, you always knew that he believed you could do better. David was brave. I know of no one of his academic stature who was subject to so many brickbats from other academicians for silly reasons. For example, David, not being an establishment investigator, studied infections when the SGI in-group thought this area was not worth investigating. "Far better to evaluate ovarian and placental enzymes—that's where the action is." He was a foreigner. Imagine a "Brit" trying to tell us Americans about science. How they misjudged this forward-thinking man. He did not know his place. Imagine an academician trying to do a scientific study on abortion in Boston. Proper scientists do not do politically unacceptable research. David loomed above all of these "stunted" academic detractors. He was an academic giant in the midst of "gnats." His legacy to science will always remain. Most important to me were his cherishing and encouragement of young investigators. As a founding member and the second president of the Infectious Disease Society for Obstetrics and Gynecology, David provided an openness and warmth for young investigators. He welcomed everyone with open arms and helped the Society expand and achieve national prominence. While always critical, he never denigrated young investigators. Rather, he pointed out missing details and left the impression that he expected more from them because they had so much potential, which is a different scene from our current Society's division into two camps, the haves and the have-nots, a setting in which the old eat their young and diminish the future of their society. David would not have stood for this. David, you are missed. I loved your counsel and your friendship. American academic obstetrics and gynecology owes you a great debt and is a lesser institution with your passing.

If David had read my article in this issue, he would have said: "Bill Ledger, you've done it again. You've managed to insult American obstetrician-gynecologists by telling them that their current level of care is not good enough and you've rubbed the noses of the American College of Obstetricians and Gynecologists into the mud by informing everyone that one of their technical bulletins is a scientific farce. Bravo, you are right. Our specialty needs to improve its care of infections in women and this will only come if we are honest enough and brave enough to question the current inadequacies of care. Let the debate begin. Let's bring American obstetrics into the 20th century."

*William J. Ledger, M.D.*
*Given Foundation Professor of Obstetrics and Gynecology*
*Cornell University Medical College*
*New York, New York*

David was a close friend of John Van S. Maeck, chairman of the Department of Obstetrics and Gynecology at the University of Vermont. I first met David in the early 1970s when he visited Vermont. We immediately identified a mutual interest in infectious diseases. From that point on, David served as one of my most cherished mentors. Frequent phone calls to discuss a paper, solicit a chapter for one of the endless books he was editing, plan a project, or just to chat were critical stimuli to my academic career. That a world-renowned academician would take time from his busy day to encourage an unknown assistant professor from Vermont was enormously empowering. His counsel was always thoughtful, candid, enthusiastic, and warm-hearted despite being delivered in his typically gruff style. Several years later, during a particularly difficult period in his career, he nonetheless found time to oversee my election to fellowship in the Infectious Disease Society of America, a major honor for an obstetrician-gynecologist at that time. I will never forget the value of his mentoring to my career nor cease to marvel at the generous and good-natured way it was given. Although I try to emulate this approach with my younger colleagues today and will never achieve David's remarkable ability as a mentor, his example is the goal to which I strive. Mentoring the younger members of our discipline is the most important task for senior academicians, and at this activity David Charles had no peer.

David was an actively practicing physician, a researcher, and an educator. It is not surprising, therefore, that he particularly enjoyed finding answers to clinical problems and making this information known to other practitioners. I think he would have approved of my article in this issue because it provides a new piece of data that may be helpful to clinicians.

I also think he would have approved of the supporting references, which I have tried to make as complete as possible. David Charles had an unbelievable knowledge of the medical literature, particularly that related to obstetric and gynecologic infectious diseases. His books and papers always contained exhaustive references, culled from the entirety of the world's scientific writing. I believe that he could have beaten any Medline search with just the information he carried about in his head.

I also imagine that David would have been dismayed by the narrow scope of this report, for he was a larger-than-life individual who tackled large projects, wrote encyclopedic works, and saw the world in its largest context.

Finally, if David had reviewed this paper, I know it would have been a thoughtful, scholarly, and careful evaluation, and I would have learned much from it. I shall greatly miss his friendship and his wisdom.

*Philip B. Mead, M.D.*
*Professor*
*Department of Obstetrics and Gynecology*
*University of Vermont College of Medicine*
*Burlington, Vermont*

Physically, David Charles was a bear of a man whose heart was larger than his body. Intellectually, he was a consummate academician who clearly understood that knowledge was a gift to be shared. He had a generosity of spirit that was reflected in the deep friendships he nourished.

I have prepared this article on medical education out of my sincerest admiration for David. It is to David Charles, the consummate intellectual, that this article on medical education is dedicated in hopes that he would have enjoyed it.

*Gilles R.G. Monif, M.D.*
*Professor*
*Department of Obstetrics and Gynecology*
*Creighton University School of Medicine*
*Omaha, Nebraska*

David Charles was not only a founding member of the Infectious Disease Society for Obstetrics and Gynecology and its second president but, in my view, the driving force that brought the Society and its members into the mainstream of the infectious-disease world. We were, at the outset, a group of clinicians with an interest in infections and antibiotics. Our early meetings were devoted almost exclusively to reports of therapeutic and prophylactic antibiotic trials, not always very well done, I might add. In his inimitable fashion, David chided, cajoled, and pressed us toward a more scientific base and the credibility of the Society was achieved.

At a personal level, David was a good friend whom I miss very much. In the early days of our friendship, he was a sage advisor whose counsel I sought and respected. His wit was often subtle, occasionally even a bit obscure, but always signalled by a loud guffaw, a wry smile, and a vigorous wringing of his hands. Some of my fondest memories of David relate to one of the Society's early meetings in Tubingen. He and I met in New York to solicit support and to plan a visit to London and the Royal College en route to Germany. Who could forget David reviewing the troops in the snow in that small German village and how much all of us enjoyed that trip. We speak of it often.

I am not sure how David would critique my review article. One thing is certain: his comments would be candid. I suspect he might comment on the fact that I have written a review article because my career has taken an administrative direction and I am no longer engaged in practice or clinical research. I also suspect that he would express strongly held views of his own on the subject of my article, for David was never one to shy away from controversy. I wish he were here to write the review. I am honored to have been invited to participate in this tribute to my wonderful friend and revered colleague, David Charles.

*Richard H. Schwarz, M.D.*
*Professor of Obstetrics and Gynecology*
*State University of New York*
*Health Science Center at Brooklyn*
*Brooklyn, New York*

